# A Clinically Translatable Multimodal Deep Learning Model for HRD Detection from Histopathology Images

**DOI:** 10.3390/diagnostics16020356

**Published:** 2026-01-21

**Authors:** Mohan Uttarwar, Jayant Khandare, P. M. Shivamurthy, Aditya Satpute, Mohit Panwar, Hrishita Kothavade, Aarthi Ramesh, Sandhya Iyer, Gowhar Shafi

**Affiliations:** 1 School of Consciousness, Dr. Vishwanath Karad MIT World Peace University, Kothrud, Pune 411038, India; jayant@actorius.co.in; 2 1Cell.Ai, 209 B, GO Square, Aundh-Hinjewadi Road Wakad, Pune 411057, Indiahrishita.kothavade@1cell.ai (H.K.);; 3 1Cell.Ai, 320, Hutch Dr, Foster City, CA 94404, USA

**Keywords:** artificial intelligence, HRD, transformers, embeddings, TRINITY, transcriptomics, clinico-molecular, PARPi

## Abstract

**Background:** With extensive research and development in the past decade, the affordability of Poly (ADP-ribose) polymerase (PARP) inhibitor therapy has drastically improved. Homologous recombination deficiency (HRD), a key biomarker, has been identified as an important guiding factor for PARP inhibitor therapeutic decisions in breast and ovarian cancer. However, identification of patients who will respond to Poly (ADP-ribose) polymerase (PARP) inhibitor therapy is challenging due to the lack of a unifying morphological phenotype. Current HRD testing via next-generation sequencing (NGS) is tissue-dependent, has high failure rates, misses relevant HRD genes, and involves longer turn-around times. **Methods:** To overcome these limitations, we developed a multimodal AI model, TRINITY, combining imaging, image-based transcriptome data, and clinico-molecular data, to examine whole-slide images (WSIs) obtained from hematoxylin and eosin (H&E)-stained samples to non-invasively predict HRD status. **Results:** The TRINITY model, tested on 316 TCGA breast and OV samples, presented a sensitivity of 0.77 and 0.91, NPV of 0.94 and 0.86, PPV of 0.63 and 0.58, specificity of 0.89 and 0.47, and AUC-ROC of 0.91 and 0.72, respectively. The model also yielded a similar outcome in a blind study of 74 samples, with a sensitivity of 81.2, NPV of 0.85, PPV of 0.77, specificity of 0.81, and high AUC-ROC value of 0.89, showing its promising preliminary evidence of predicting HRD status on external cohorts. **Conclusions:** These findings demonstrate TRINITY’s potential as a rapid, cost-effective, and tissue-sparing alternative to conventional NGS testing. While promising, further validation is needed to establish its generalizability across broader cancer types.

## 1. Introduction

The integration of artificial intelligence (AI) into histopathology has revolutionized cancer diagnostics, enabling the extraction of intricate morphological and spatial features from hematoxylin and eosin (H&E)-stained whole-slide images (WSIs) [1]. AI-driven approaches have demonstrated remarkable potential in predicting molecular phenotypes and genomic alterations from histopathological images. Various AI models have been successfully applied to classify cancer subtypes, predict microsatellite instability (MSI) status, and infer tumor mutational burden (TMB) from WSIs [2,3]. These advancements underscore the transformative role of AI in bridging the gap between histopathology and molecular diagnostics.

Stratifying the patients who will respond to PARP inhibitors remains a complex task due to the limitations of current biomarkers and testing methodologies. While BRCA1 and BRCA2 mutations are well-established indicators of homologous recombination deficiency (HRD), not all patients with HRD respond equally to PARP inhibition. Conversely, some patients without BRCA mutations still exhibit sensitivity, highlighting the need for broader and more accurate markers of DNA repair deficiency. Current HRD tests, such as genomic scar assays, reflect historical, not functional, DNA repair status, often leading to false positives or negatives [4,5]. The lack of standardized thresholds and inconsistent sensitivity across tumor types further complicates clinical decision-making.

Tumor heterogeneity adds another layer of complexity in predicting PARP inhibitor response. Within a single tumor, subpopulations of cells may differ in their DNA repair capabilities, resulting in partial or transient responses to therapy [6]. Moreover, the HRD status of a tumor can evolve over time due to selective pressures, including prior chemotherapy, potentially restoring homologous recombination through mechanisms like BRCA reversion mutations [6,7,8]. These dynamic changes are not captured by one-time genomic assays, underscoring the need for real-time or functional assessments of DNA repair activity, such as RAD51 foci formation assays, which are not yet widely accessible in routine clinical practice [9].

Clinical and logistical barriers also impede accurate diagnosis of PARP inhibitor responders [10,11]. Many patients do not have access to comprehensive molecular testing due to cost, lack of infrastructure, or insurance constraints. Additionally, germline testing alone may miss relevant somatic mutations, and many detected alterations are classified as variants of uncertain significance, providing little actionable guidance. Even when genetic alterations are identified, emerging resistance mechanisms—such as alternative DNA repair pathway activation or epigenetic changes—may limit the long-term efficacy of PARP inhibitors [12]. Overcoming these challenges requires the development of standardized, functional, and widely deployable diagnostic tools to better guide patient selection and improve treatment outcomes.

Recent studies have highlighted the ability of AI to detect subtle visual markers in H&E-stained WSIs, which correlate with underlying genomic alterations and molecular phenotypes [13,14,15]. This capability is particularly relevant for homologous recombination deficiency (HRD) prediction, as HRD arises from complex genomic and molecular mechanisms that manifest in cellular morphology. By training AI models on H&E-stained WSIs, researchers have achieved high-precision predictions of molecular status, offering a non-invasive and cost-effective alternative to traditional methods [15]. Moreover, AI-driven digital pathology enables the integration of multi-omics data, enhancing the accuracy of predictive models and supporting personalized therapeutic strategies [16].

HRD has emerged as a critical biomarker in oncology, guiding therapeutic decisions for Poly (ADP-ribose) polymerase (PARP) inhibitors and platinum-based treatments. HRD reflects genome-wide scarring and impaired DNA repair mechanisms, often resulting in chromosomal instability [17]. Traditional methods for HRD detection, such as next-generation sequencing (NGS), are highly effective but limited by their cost and lengthy turnaround times [18,19]. These limitations have spurred the exploration of alternative approaches, particularly those leveraging advancements in digital pathology and AI.

It is known that HRD is caused by BRCA1/2 mutations and yet not all patients with mutations in BRCA1/2 respond to PARP inhibitors [20,21]. The differences in response to PARP inhibitor treatment could also result from the development of resistance. In addition, PARP inhibitor resistance could result from restoration of homologous recombination (HR) repair due to secondary mutations in BRCA1/2 and depletion of HR compensatory repair pathways such as the non-homologous end joining pathway [22].

This study demonstrates the application of an AI-driven multi-omics model combining three different modalities of data, viz., images, AI-inferred transcriptomic data, and clinico-molecular data, forming the TRINITY model for HRD screening in patients with breast and ovarian cancers. Its superior performance characteristics highlight its potential clinical utility in accurately identifying candidates for PARP inhibitor therapy and platinum-based chemotherapy, thereby enabling more effective treatment selection with reduced toxicity.

## 2. Materials and Methods

### 2.1. Study Cohorts

**Figure 1:**
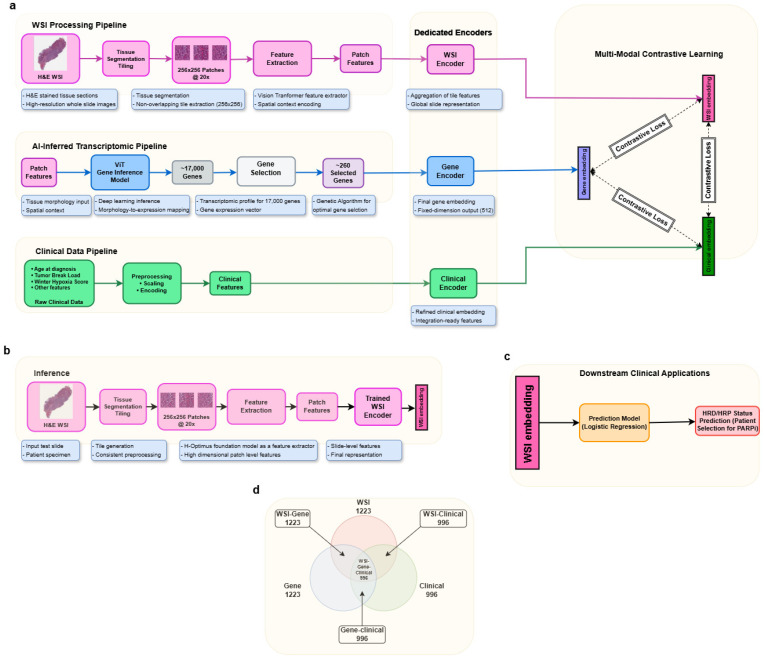
Illustrates the overall framework, highlighting the (**a**) training workflow of TRINITY AI using multimodal data integration, (**b**) inference pipeline based on the multimodal features extracted from given WSI under study, (**c**) HRD/HRP status prediction using an ML model trained on a combined BRCA-OV cohort of 807 patients finally to select the patients for PARPi treatment, and (**d**) distribution of patients across data modalities with 996 patients forming the complete training cohort used for TRINITY model optimization.

A combined cohort of 1223 patients from The Cancer Genome Atlas (TCGA) encompassing both breast and ovarian cancer samples were considered for the model development. To ensure the integrity and consistency of multimodal training, only patients with complete molecular, morphological, and AI-inferred transcriptomic data were included. This yielded a final training dataset of 996 patients to train a transformer-based multimodal AI model. This yielded 512 representative features in the form of embeddings, which are used as the feature set to further train an ML model, typically a Gradient Boost model, which performs the function of classifier. The classifier model was trained using a training set of 807 patients and a validation cohort of 343 patients drawn from the harmonized dataset. Figure 1a shows the complete workflow of building the TRINITY-based HRD/HRP status prediction model. Figure 1b,c show the two stages of the inferencing process using the model. The multimodal dataset used for building the model is shown in Figure 1d.

### 2.2. Data Modality Acquisition and Preprocessing: The TRINITY AI Multimodal Architecture

The TRINITY architecture unifies three complementary modalities, including morphology (WSI-based), molecular/pathological, and AI-inferred transcriptomics, into a single integrated representation for each patient sample to infer HRD/HRP status. The main idea of multimodal model building is from CLIP [23]. A similar approach has been adapted in the field of pathology in m-STAR [24].

Each modality was independently processed through a dedicated encoder:

- Morphological features used a Vision Transformer embedding network.
- Molecular/pathological features were processed via a multilayer perceptron (MLP) encoder.
- AI-inferred transcriptomic features were encoded using an MLP optimized for high-dimensional biological data.

Each encoder produced a fixed-length embedding vector, forming modality-wise representations.

### 2.3. Molecular Data

Molecular data formed one of the three core inputs for the feature selection framework (TRINITY AI). For all samples, structured molecular and pathological data were obtained from TCGA repositories. Disease stage distribution and available demographic characteristics for both breast cancer (TCGA-BRCA) and ovarian cancer (TCGA-OV) cohorts are presented in Table 1.

Essential variables were selected for their relevance to HRD biology and included genomic event (GE) burden, ICD histology, aneuploidy score, TIMEx features capturing the tumor immune microenvironment, and three mutational signatures specifically linked to homologous recombination deficiency (HRD) [25,26,27].

To ensure data consistency, we applied standardized preprocessing across all variables. Numerical features (e.g., aneuploidy score) were normalized, while categorical attributes (such as ICD histology codes) were one-hot encoded [27]. Adhering to a complete-case analysis, we retained only patients with all features available across modalities, ensuring robustness in integrative modeling [28]. This approach maintained the interpretability of clinical signals without introducing imputation-based artifacts.

### 2.4. Digital Pathology (WSI) Processing and Morphological Feature Extraction

For the morphological modality, we processed hematoxylin and eosin (H&E)-stained whole-slide images (WSIs) using a dedicated image preprocessing pipeline. The HistoQC [1] (https://github.com/choosehappy/HistoQC accessed on 15 January 2026) algorithm was employed to segment tissue from background and artifacts, ensuring that only biologically relevant regions were analyzed.

Each WSI was divided into non-overlapping 256 × 256-pixel patches at 20× magnification. The H-Optimus foundation models [29] (Bioptimus (accessed via Hugging Face Hub, 2024)) used for extracting the H&E embeddings. It is an advanced multimodal, multi-scale AI model for histology developed by Bioptimus. It is trained on over one million whole-slide images from more than 800,000 patients and achieves state-of-the-art performance across key pathology tasks. Since the foundation Vision Transformer model H-Optimus used for representation extraction was pre-trained on extensively heterogeneous staining profiles, no color normalization was applied. The extracted patch-level embeddings were aggregated into slide-level feature bags, representing the morphological phenotype of each tumor sample. These morphological feature sets served as one of the three primary modalities fed into the TRINITY multimodal encoder.

### 2.5. AI-Inferred Transcriptomic Feature Generation

To generate transcriptomic profiles directly from histopathology images, we utilized a Vision Transformer (ViT) network trained on paired H&E images and RNA-seq data from the same samples. The model predicted gene expression profiles across approximately 17,000 genes for each sample, reaching a median Pearson correlation coefficient (PCC) of 0.6061 with respect to ground-truth expression data from RNA sequencing.

A three-step gene selection pipeline was implemented to reduce dimensionality and retain biologically meaningful signals:

1. Differential expression analysis identified genes associated with HRD/HRP classification [30].
2. Genes were then filtered based on their correlation between predicted and actual transcriptomic values.
3. A genetic algorithm searched for the optimal subset of genes exhibiting maximal predictive synergy [31].

This process yielded a final curated panel of 260 genes, forming the AI-inferred transcriptomic feature input for the TRINITY model.

### 2.6. Contrastive Alignment in a Shared Latent Space

To achieve cross-modal semantic integration, modality-specific embeddings were projected into a shared latent space through contrastive learning [24]. The objective function encouraged embeddings from the same patient—across different modalities—to align closely, while maintaining separation from other patients’ representations. This approach leveraged an InfoNCE loss formulation, fostering complementary representation of learning across clinico-molecular, morphological, and transcriptomic signals [32].

### 2.7. The Final TRINITY Embedding

Out of a combined cohort of 1223 breast and ovarian cancer samples, multimodal data from 996 samples were used to train the TRINITY model. The outcome of this training is a unified TRINITY embedding a cross-modal vector representation encapsulating genomic, histopathologic, and clinico/molecular signatures relevant to homologous recombination biology. These embeddings form the input to the machine learning classifier, enabling high-fidelity HRD/HRP status prediction to guide potential PARP inhibitor response stratification in breast and ovarian cancer patients.

### 2.8. ML Model Training and Testing Methodologies

The dataset comprised 1150 samples from The Cancer Genome Atlas (TCGA) and 74 samples from an external accredited laboratory cohort, representing both breast cancer (BRCA) and ovarian cancer (OV) subtypes as shown in Table 2.

The samples were further stratified into HRD-High and HRD-Low categories. Specifically, the TCGA cohort contributed to 145 HRD-High and 593 HRD-Low BRCA cases and 36 HRD-High and 33 HRD-Low OV cases for training. The test set from TCGA included 62 HRD-High and 254 HRD-Low BRCA samples, along with 12 HRD-High and 15 HRD-Low OV samples. Additionally, the external cohort, used exclusively for independent testing, consisted of 32 HRD-High and 42 HRD-Low OV samples. This distribution ensured robust cross-cohort and cross-cancer evaluation to assess model generalization. Model training was performed using a Gradient Boosting Classifier, optimized through systematic hyperparameter tuning to balance model complexity and generalization capability [33]. All data splits (training and testing) were performed in a stratified manner to preserve the original HRD-High/HRD-Low class proportions within each cohort, thereby mitigating the impact of class imbalance on performance estimates.

In addition, baseline performance comparison of the classification models prior to hyperparameter tuning are presented in Table 3. Baseline results reflect default configurations and do not necessarily capture a model’s capacity to adapt to complex, heterogeneous feature spaces after optimization. Gradient Boosting was selected for hyperparameter tuning because it is particularly well suited to the characteristics of our learned multimodal embeddings. Specifically, the embeddings integrate non-linearly interacting features derived from histopathology, transcriptomics, and clinical data. Gradient Boosting models iteratively refine decision boundaries by correcting residual errors, enabling them to capture subtle, higher-order feature interactions that are difficult for linear models and kernel-based methods to exploit effectively in this setting. While models such as SVC showed strong baseline performance, their gains after tuning were limited due to sensitivity to kernel choice and scaling in relatively small, imbalanced clinical datasets. In contrast, Gradient Boosting benefited substantially from tuning key hyperparameters (e.g., learning rate, tree depth, and number of estimators), resulting in improved generalization and a more favorable sensitivity–specificity balance on the held-out validation and external test cohorts.

### 2.9. Model Configuration and Hyperparameter Optimization

A Gradient Boosting Classifier was employed to predict homologous recombination deficiency (HRD) status from multimodal feature representations. Gradient Boosting is an ensemble learning technique that constructs multiple weak learners, typically decision trees, in a sequential manner. Each subsequent tree attempts to correct the errors made by its predecessors, thereby minimizing the overall loss function and enhancing predictive performance. This iterative boosting mechanism allows the model to capture complex, non-linear relationships inherent in high-dimensional biomedical data such as genomic and histopathological features. To ensure optimal model performance and generalization, an extensive Grid Search hyperparameter optimization was conducted [25]. Grid Search systematically evaluates all possible combinations of predefined hyperparameter values using cross-validation to identify the configuration that maximizes predictive accuracy and stability. In this study, key parameters such as the learning rate, maximum tree depth, number of estimators, subsampling ratio, and feature selection strategy were tuned through this approach (Table 4). The final optimized configuration employed a learning rate of 0.01, ensuring gradual and stable convergence during training, and a maximum depth of 7, allowing the model to capture intricate data structures while controlling overfitting. The maximum number of features considered at each split was defined as log_2_(n_features) to promote diversity among trees and reduce correlation between them. The minimum samples per leaf and minimum samples per split were set to 1 and 5, respectively, to maintain model granularity without creating overly specific branches. Furthermore, 100 boosting estimators were used to balance predictive strength and computational efficiency, and a subsample ratio of 0.8 was applied to introduce randomness and enhance robustness. This carefully tuned Gradient Boosting framework demonstrated strong discriminative capability and consistent generalization across training and independent testing cohorts, confirming its suitability for HRD classification in the cancer domain.

### 2.10. Data Leakage Prevention During Training

We implemented strict data compartmentalization and isolation strategies, as detailed below.

1. Patient-level separation: All data (imaging, transcriptomic, and molecular) from the same patient were exclusively assigned to a single split (training, validation, or external test). No patient data appeared across multiple sets.
2. Modality-specific preprocessing isolation:
  - WSI preprocessing (including HistoQC filtering and patch extraction) was performed independently within each data split, without sharing statistics or parameters across splits.
  - Gene selection using differential expression analysis and a genetic algorithm was conducted only on the training set, and the resulting gene panel was subsequently applied unchanged to the validation and test sets.
3. Multi-stage pipeline safeguards:
  - Stage 1 (multimodal embedding via contrastive learning):
    The multimodal embedding framework was trained using a contrastive learning paradigm, which does not rely on HRD ground-truth labels. Instead, the model learns a shared embedding space by pulling semantically similar (positive) cross-modal pairs closer together while pushing dissimilar (negative) pairs farther apart. Since no outcome labels are used at this stage, and training is restricted to the training split, this design inherently reduces the risk of label leakage while preserving strict data separation.
  - Stage 2 (Gradient Boosting Classifier):
    Used pre-generated embeddings from Stage 1, ensuring no information flow back to embedding generation.
  - Hyperparameter tuning:
    Hyperparameter optimization for the Gradient Boosting Classifier was performed using stratified cross-validation within the training set only. A completely held-out validation set was used solely for final model selection, without participation in training or tuning.
4. No data reuse: The 260-gene panel selected during training was not refined on validation/test data. Clinical variables used for molecular encoder were not selected based on validation set correlation. This is completely based on the training set.
5. Independent test set: The external cohort (n = 74) was never viewed during model development, training, or hyperparameter selection and was used for blind evaluation only.

## 3. Results and Discussion

The TRINITY model, trained on the TCGA breast and ovarian cancer datasets and predicted on the collated datasets of TCGA-(breast and ovarian) (n = 343), TCGA-breast (n = 316), and TCGA-ovarian (n = 27), yielded an overall sensitivity of 79%, 77%, and 91% respectively. The model specificity, PPV, and NPV values are shown in Table 5.

Table 5 shows performance assessments of the TRINITY model on breast and ovarian cancer samples. Predictive performance was assessed using sensitivity, specificity, PPV, NPV, and AUC.

**Figure 2:**
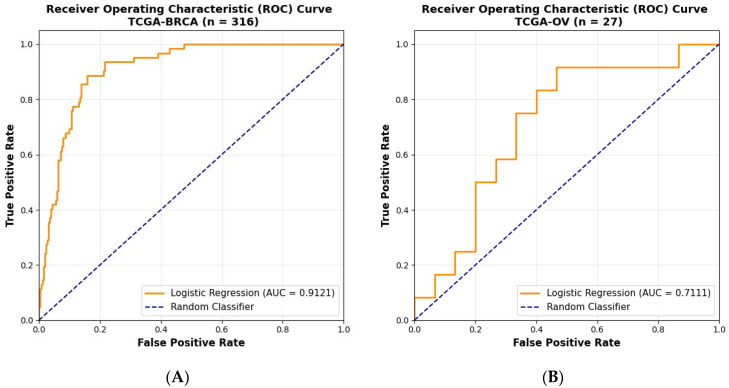

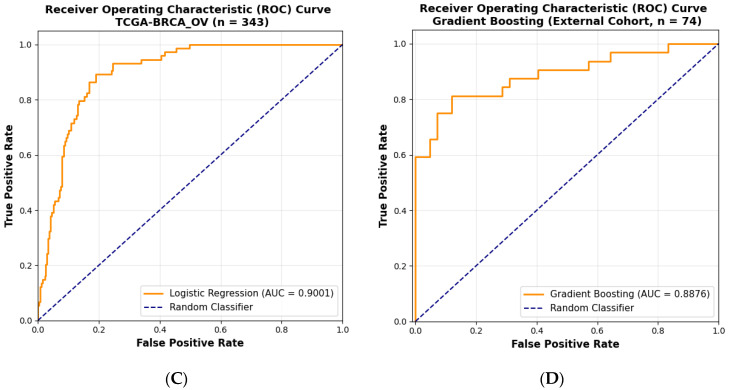
AUC-ROC curves obtained for (**A**) TCGA-breast (**B**) TCGA ovarian, (**C**) TCGA-breast, and TCGA-ovarian cancers combined and (**D**) External cohort.

The AUC-ROC values evaluated for the model for test datasets are 0.90, 0.91, 0.71, and for external cohort, 0.89, respectively, which are also shown in Figure 2. The best AUC-ROC value was obtained for the TCGA-breast cancer samples (0.9121) showing its ability of superior classification. The model effectively captures complex genomic signatures beyond traditional single-gene alterations such as BRCA1/2 mutations. By integrating diverse features, including morphological, molecular, and pathological signatures, the model improves upon conventional rule-based HRD classifiers that may miss borderline or atypical cases [34].

The NPV of the TCGA-breast and TCGA- breast and ovarian datasets predicted using the TRINITY model shows the capability of our AI model to effectively screen HRD-negative patients. Clinically, this could be promising in carefully excluding HRD-negative patients for PARPi and platinum-based therapy. The model utilized in this study shows better performance for breast and ovarian cancers when compared to several similar working systems [35]. This low-cost, rapid detection approach can support the prioritization of tissue samples for confirmatory molecular assays and facilitate the identification of patient subsets who may not benefit from approved therapies or meet eligibility criteria for clinical trial enrollment.

The AUC-ROC of our model outperforms, as compared with other published works, as shown in Table 6.

Key differences elaborated from Table 6.

1. Data scope: DeepHRD and Kather Lab used broader multi-institutional cohorts; our TCGA-centric approach limits direct comparison but provides internal harmonization benefits.
2. Modality differences: DeepHRD and Kather Lab rely on morphology alone; TRINITY uniquely integrates imaging, AI-inferred transcriptomics, and clinical data. This multimodal approach explains performance advantages but reflects different clinical utility (more data-intensive).
3. Task definition: All three methods predict HRD status, but TRINITY additionally incorporates clinico-molecular data (BRCA status, aneuploidy, and mutational signatures), making it more clinically actionable for patient stratification.
4. Evaluation metrics: We now report all methods using consistent metrics (sensitivity, specificity, AUC, PPV, and NPV) rather than selective reporting.
5. Generalization: We acknowledge that our external validation (n = 74 ovarian) shows AUC = 0.88, directly comparable to Kather Lab’s 0.61 on external OV cohorts, suggesting improvement. However, we note their 10-cancer-type validation is broader in scope.

Importantly, the high sensitivity obtained from the model also ensures that fewer HRD-positive patients are overlooked, thereby expanding access to targeted therapies and improving patient selection for clinical trials evaluating DNA damage–response agents.

To further progress through its clinical utility, the model warrants larger cohort training and testing. However, this AI-driven HRD detection approach represents a promising advancement toward scalable, accurate, and broadly accessible genomic stratification. Its integration into clinical workflows has the potential to refine therapeutic decision-making and enable more precise identification of patients who may benefit from HRD-targeted interventions. Because the AI model can perform well even with low tumor fraction or limited sequencing data, more patients, especially those with small biopsies, can be evaluated for HRD and considered for PARPi treatment.

### 3.1. Cost-Effectiveness and Turnaround Time Advantages

TRINITY offers substantial practical advantages over current HRD testing methods. The estimated cost per test would primarily be attributable to digital slide scanning and computational processing, compared to $3000–5000 for superior HRD testing methods in the market. This represents a considerable cost reduction, with potential for further reduction as digital pathology infrastructure becomes standard [37].

The turnaround time for TRINITY is approximately 24 h from digital slide availability (or 2–3 days, including slide scanning), compared to 21 days for global HRD testing methods. This rapid turnaround enables timely treatment decisions, particularly important in the neoadjuvant setting where treatment planning must occur quickly after diagnosis [38].

### 3.2. Limitations and Future Directions

This study has several limitations that warrant consideration. First, the training data come primarily from TCGA, which represents high-quality research-grade specimens that may not fully reflect real-world clinical samples with variable fixation, processing, and staining quality. Validation on diverse multi-institutional cohorts partially addresses this concern, but prospective evaluation in routine clinical settings is needed.

Second, while TRINITY demonstrates excellent cross-sectional accuracy for HRD status, longitudinal prediction of acquired resistance to PARP inhibitors remains unexplored. Future studies should evaluate whether sequential TRINITY assessments on pre-treatment and post-progression biopsies can detect HRR restoration and predict secondary resistance. Integration with liquid biopsy approaches could enable non-invasive monitoring.

Third, the current study focused on breast and ovarian cancers, where HRD is well-established. Expansion to other cancer types (pancreatic, prostate, or endometrial) where HRD is emerging as a therapeutic target requires additional validation. The transferability of TRINITY across cancer types through transfer learning should be investigated.

Finally, while the external cohort (n = 74) demonstrates strong performance metrics, validation on larger, multi-institutional datasets is essential before clinical implementation. This study should be considered as “hypothesis-generating” for broader applicability.

### 3.3. Broader Impact and Future Research Directions

TRINITY exemplifies the potential of multimodal AI to transform precision oncology by integrating diverse data types into clinically actionable predictions. The contrastive learning framework used in TRINITY is broadly applicable to other biomarker prediction tasks: microsatellite instability, tumor mutational burden, PD-L1 expression, and emerging targets for immunotherapy and targeted therapy.

The 260-gene signature developed for TRINITY provides biological insights into HRD phenotypes beyond BRCA mutations. Pathway analysis and functional validation of high-importance genes (particularly APOBEC3B, CXCL13, and ESR1) may reveal novel therapeutic targets and resistance mechanisms. The integration of single-cell RNA-seq data could further refine understanding of HRD heterogeneity within tumors.

1. Gene Ontology enrichment analysis: Top genes from the 260-gene panel were analyzed for functional categories. Enriched terms include the following:
  - DNA damage response (*p* < 0.001): ATM, CHEK2, TP53BP1, and RAD51 pathway members
  - Homologous recombination repair (*p* < 0.001): BRCA1, BRCA2, RAD51D, PALB2, and BARD1.
  - Apoptosis regulation (*p* < 0.01): BAX, BAD, and TP53 targets
2. Highlighted clinically actionable genes: a.APOBEC3B: High expression in HRD-High samples; implicated in PARP inhibitor sensitivity and genomic instability b.CXCL13: Immune infiltration marker; elevated in HRD-High, suggesting immunogenic phenotype c.ESR1: Interaction with HR repair pathway; explains differential response in ER+ breast cancers
3. AI prediction validation: Median Pearson correlation coefficient (PCC = 0.6061) between AI-predicted and true expression is discussed in context of recent literature.
4. Limitations acknowledged: We note that while genes are enriched for HRD biology, some selected genes may reflect tumor microenvironment or immune context rather than intrinsic HR deficiency, suggesting future work should integrate single-cell transcriptomics.

Expansion of TRINITY to predict treatment response (not just HRD status) represents a logical next step. Training on outcome data (progression-free survival, overall survival, and treatment response) could enable direct prediction of PARP inhibitor benefit, potentially improving patient selection beyond binary HRD classification. Integration with pharmacogenomic data could enable personalized dosing and toxicity prediction.

The contrastive learning framework could be extended to incorporate additional modalities: immunohistochemistry (RAD51 foci and γ-H2AX), spatial transcriptomics, proteomic data, and radiologic imaging (CT, MRI, or PET). True multi-omics integration capturing complementary information across all biological levels could further improve accuracy and biological insight.

Finally, federated learning approaches could enable TRINITY training across multiple institutions without sharing patient data, addressing privacy concerns while leveraging larger, more diverse datasets. This would be particularly valuable for rare cancer subtypes and underrepresented populations where single-institution datasets are limited. The complete implementation was carried out in a Python (version 3.11.9) environment using PyTorch (version 2.3.1) as the primary deep learning framework, along with additional supporting Python libraries. In particular, the gradient boosting model was implemented using Scikit-learn (version 1.3.2). This software stack improves the extensibility and portability of the system, enabling the research to be translated into a user-friendly, scalable product suitable for deployment as an effective clinical utility.

## 4. Conclusions

In conclusion, the prediction of HRD using AI-based models has emerged as a promising advancement in precision oncology. This study highlights the potential of AI-powered histopathological image analysis as a rapid, non-invasive screening tool for HRD screening. These models aim to provide scalable, real-time solutions that can complement or even substitute for molecular assays in certain clinical settings. The strong performance of the ovarian and breast cancer-specific models, coupled with the model’s high specificity, underscores its clinical utility in optimizing patient stratification for HRD-targeted therapies such as PARP inhibitors and platinum-based treatments. While challenges related to tumor heterogeneity remain, our findings provide a foundation for further refinement and expansion of this framework. Future work necessitates validating the model on larger and more diverse pan-cancer datasets, as well as exploring its applicability to other clinically actionable molecular layers, including transcriptome and clinical features. By advancing the role of AI in precision oncology, there is immense potential to transform cancer diagnostics and therapeutic decision-making, ultimately improving patient outcomes.

## Figures and Tables

**Table 1:** Breakdown of TCGA cohort and sample demographics.

Cancer Type	Stages	Race	Ethnicity
BRCA (n = 1117)	Stage I (174) Stage II (640) Stage III (261) Stage IV (20) Unknown (22)	White (783) Black or African American (175) Asian (61) American Indian or Alaska Native (1) Not Available (97)	Not Hispanic or Latino (908) Hispanic or Latino (40) Not Available (169)
OV (n = 106)	Stage I (2) Stage II (4) Stage III (74) Stage IV (23) Unknown (3)	Not Available (106)	Not Hispanic or Latino (93) Hispanic or Latino (2) Not Available (11)

**Table 2:** Dataset composition.

Cohort	Cancer Type	Training (HRD-High)	Training (HRD-Low)	Validation (HRD-High)	Validation (HRD-Low)	External (HRD-High)	External (HRD-Low)	Total
TCGA	BRCA	145	593	62	254	–	–	1054
		738		316				
	OV	36	33	12	15	–	–	96
		69		27		–	–	
	BRCA + OV	738 + 69 = 807 (Total Training)		316 + 27 = 343 (Total Testing)		–	–	1150
External	OV	–	–	–	–	32	42	74

**Table 3:** Baseline performance comparison of classification models prior to hyperparameter tuning.

Model	AUROC	Accuracy	Sensitivity (Recall)	Specificity	PPV (Precision)	NPV
Logistic Regression	0.5917	0.5319	0.8750	0.3548	0.4118	0.8462
Random Forest	0.6129	0.5213	0.8125	0.3710	0.4000	0.7931
Support Vector Classifier	0.6436	0.6383	0.6562	0.6290	0.4773	0.7800
Gradient Boosting	0.5766	0.5213	0.7500	0.4032	0.3934	0.7576
K-Nearest Neighbors	0.5393	0.4149	0.8750	0.1774	0.3544	0.7333
Decision Tree	0.6174	0.5851	0.7188	0.5161	0.4340	0.7805

**Table 4:** Training parameters obtained from an extensive Grid Search.

Parameter	Value	Description
learning_rate	0.01	Controls how much the model updates its weights after each iteration; smaller values make training slower but stable.
max_depth	7	Sets the maximum depth of each decision tree, allowing the model to capture complex relationships while controlling overfitting.
max_features	log2	Determines the number of features to consider when splitting a node, helping to reduce overfitting by introducing feature randomness.
min_samples_leaf	1	Ensures that each terminal leaf node contains at least one training sample, allowing fine-grained decision boundaries.
min_samples_split	5	Specifies the minimum number of samples required to split an internal node, preventing the creation of overly specific branches.
n_estimators	100	Sets the total number of boosting stages (trees) to be built; more estimators can improve performance but increase computation.
subsample	0.8	Uses 80% of the training data for each tree to introduce randomness and improve the model’s generalization.

**Table 5:** The predictive performance of TRINITY-HRD as assessed based on various metrics and different cohorts.

Model Predict on	Total Samples	Sensitivity	NPV	PPV	Specificity	AUC ROC
TCGA- breast and ovarian	343	0.7973	0.9395	0.6211	0.8662	0.9001
TCGA-breast	316	0.7742	0.9417	0.6316	0.8898	0.9121
TCGA-ovarian	27	0.9167	0.875	0.5789	0.4667	0.7111
External validation set	74	0.812	0.854	0.765	0.81	0.888

**Table 6:** Comparison of the AUC-ROC achieved by TRINITY-HRD with values reported in existing literature.

Source		Modal	Data Source	BRCA AUC	OV AUC	Key Differences
DeepHRD [14]		Image	TCGA	0.81	-	ResNet18 CNN; no multimodal integration
Kather Lab HRD [36]		Image	Multi-cohort	0.78	0.61	Attention- based MIL; single modality; 10 cancer types tested
Trinity-HRD	TCGA	Image + Gene + Clinical	TCGA	0.9121	0.7111	Integrated multimodal; contrastive learning;
				0.9001 (BRCA-OV)		
	External cohort			-	0.88	

## Data Availability

The dataset supporting the conclusions of this article will be available upon reasonable request to the corresponding authors.

## Abbreviations

The following abbreviations are used in this manuscript:

AI	Artificial intelligence
HRD	Homologous recombination deficiency
TAT	Turn-around time
H&E	Hematoxylin and eosin
TCGA	The Cancer Genome Atlas
HRR	Homologous recombination repair
AUC	Area under curve
PPV	Positive predictive value
NPV	Negative predictive value
WSI	Whole-slide image
PARP	Poly (ADP-ribose) polymerase
NGS	Next-generation sequencing
NMD	No mutations detection
HRP	Homologous recombination proficient
QC	Quality control
DL	Deep learning
